# Household Disinfection Interventions to Prevent Cholera Transmission: Facilitators, Barriers, Training, and Evidence Needs

**DOI:** 10.4269/ajtmh.20-1314

**Published:** 2021-07-06

**Authors:** Camille Heylen, Cawo Ali, Karin Gallandat, Daniele Lantagne, Gabrielle String

**Affiliations:** 1 Department of Civil and Environmental Engineering, Tufts University, Medford, Massachusetts;; 2 Department of Disease Control, London School of Hygiene and Tropical Medicine, London, United Kingdom

## Abstract

There are two common household disinfection interventions to prevent interhousehold transmission of cholera: household spraying, whereby a team disinfects cholera patients’ households, and household disinfection kits (HDKs), whereby cleaning materials are provided to cholera patients’ family members. Currently, both interventions lack evidence, and international agencies recommend HDK distribution; however, household spraying remains widely implemented. To understand this disconnect, we conducted 14 key informant interviews with international and national responders and a study in Haiti assessing HDK efficacy using two training modules including 20 household surveys and 327 surfaces samples before and after cleaning. During interviews, 80% of the international-level informants discussed evidence gaps for both interventions, and 60% preferred HDKs. Conversely, no national-level informants knew what an HDK was; therefore, they all preferred spraying. Informants discussed behavior changes, bleach perceptions, and implementation as facilitators and/or barriers to implementing both interventions. In households, training with demonstrations regarding the use of HDK led to increased reductions of *Escherichia coli* (*P* < 0.001) and *Vibrio* spp. (*P* < 0.001) on surfaces after participants cleaned the household compared with a hygiene promotion session only. These results emphasize the gap between the current international-level policy and the realities of cholera response programs, highlight the need for evidence to align household disinfection recommendations, and underscore the importance of the dissemination and training of responders and affected populations regarding methods to prevent intrahousehold cholera transmission.

## INTRODUCTION

Cholera is a diarrheal disease caused by infection with the bacterium toxigenic *Vibrio cholerae* O1/O139, which can cause profuse watery stool and vomiting, resulting in dehydration and death if untreated.^1^^,^^2^ In 2018, 34 countries reported 499,447 cholera cases with a 0.6% case fatality rate.^3^ However, this number might not capture the true disease burden.^4^

Although the ingestion of contaminated food or water is considered the primary transmission routes for cholera, there is growing evidence suggesting that person-to-person transmission within households (via contaminated objects or direct contact) might also have a role.^5^^–^^7^ Studies suggest that individuals living within 50 meters of a cholera case are 23- to 56-times more likely to contract cholera than those further away,^8^ and the mean infection risks attributable to household contacts of cases are 2- to 100-times that associated with noncontacts.^8^^,^^9^ Therefore, although long-term solutions to prevent cholera include providing adequate drinking water, sanitation, and hygiene (WASH) services, infection control interventions, such as household disinfection, are recommended to interrupt cholera transmission within the household.^10^ These interventions can be integrated with a broader rapid response strategy such as case area-targeted response (CATI).^11^^,^^12^

During cholera outbreaks, there are, to our knowledge, two current interventions for household disinfection: household spraying and household disinfection kit (HDK) distribution. According to a systematic review of WASH interventions in response to outbreaks, no evidence of the effectiveness of household spraying or HDKs to reduce *V. cholerae* was identified.^13^

Household spraying is a commonly implemented intervention whereby a team is sent to disinfect cholera patients’ homes with chlorine.^13^^,^^14^ Recently, there has been limited evidence that household spraying can reduce *V. cholerae* on household surfaces.^15^ However, implementation of household spraying has been questioned because of the lack of evidence regarding its epidemiological impact, risk of stigmatization, and logistical constraints.^10^ Despite these concerns, and despite the fact that the intervention is explicitly not recommended by four international agencies,^16^ household spraying remains widely implemented as an outbreak response.^15^

Alternatively, HDKs are distributed (sometimes with training) and contain cleaning materials for household members living with cholera patients to complete disinfection themselves.^10^^,^^17^^,^^18^ HDKs are distinct from other kits (e.g., hygiene kits, menstrual hygiene kits, shelter kits) because they specifically focus on providing materials to complete surface disinfection (including bleach) to reduce cholera transmission. To our knowledge, only one study conducted a follow-up survey after the distribution of HDKs along with health promotion sessions for cholera prevention.^19^ This study concluded that hands-on hygiene promotion sessions conducted in a purpose-built example household (along with HDK distribution) were time-consuming but necessary. During this study, 97.6% of recipients self-reported that they used the HDK contents, with > 75% self-reporting using five or more items; no evidence of disinfection effectiveness with HDK distribution was collected and reported. Despite the lack of additional evidence of the effectiveness of HDKs, international agencies suggest focusing resources on HDK distribution rather than household spraying for household disinfection.^16^^,^^20^

Overall, there is no consensus regarding the implementation of household disinfection interventions to prevent cholera transmission.^21^ To provide evidence of these interventions, we developed two studies to test the efficacy and effectiveness of household disinfection. First, we tested the laboratory efficacy of spraying and wiping various chlorine concentrations on household surfaces by replicating the disinfection mechanisms of household spraying and HDKs.^22^ Second, we intended to conduct mixed methods field evaluations (including key informant interviews [KIIs], observations, household surveys, and surface sampling) of household spraying and HDK implementation. We did evaluate household spraying interventions; three household spraying programs were evaluated to understand the effectiveness of the intervention and the results have been previously published.^15^ However, from 2017 to 2020, we were not able to identify any HDK programs for evaluation because organizations were not implementing this intervention. Therefore, we pivoted the planned HDK field evaluation portion of our research and instead conducted research to understand why HDK programs were not being implemented despite international guidance to discontinue household spraying in favor of HDK programming.

Specifically, the objectives of this work were to compare and contrast household spraying and HDK programs by understanding the implementer’s current knowledge of household disinfection interventions, successes, lessons learned, barriers, and recommendations for household disinfection interventions, and to inform responders about practices for HDK recipient training (identified as a potential barrier) by evaluating the efficacy of different pilot training modules for household members using an HDK to clean household surfaces.

## METHODS

We conducted KIIs regarding household disinfection and a pilot study in Haiti to assess the efficacy of training modules for HDK use. This work was approved by the Social, Behavioral, and Educational Research Institutional Review Board (SBER IRB) at Tufts University (#1901020 and #1903002) and the Haitian National Bioethics Committee (#1819-42).

### Key informant interviews.

A 26-question, semi-structured KII guide was developed, including sections about interviewee experiences, decision-making for WASH interventions during humanitarian emergencies and cholera outbreaks, decision-making for household disinfection intervention implementation (including household spraying and HDK), and experiences with implementing household disinfection (including successes, lessons learned, suggestions, and other comments).

E-mails soliciting participants and requesting the recipients to forward the message widely were sent to the Global WASH Cluster mailing list, the Global Task Force on Cholera Control mailing list, and personal contacts. Detailed study participation information and informed consent procedures were e-mailed to individuals responding to these general e-mails. After informed consent was obtained, interviews were conducted in English or French by Webex (San Jose, CA), Skype (Palo Alto, CA), or telephone and recorded using Webex or Quicktime Player (Cupertino, CA). Informants could also respond in writing (e.g., e-mail) upon request.

Interviews recorded in English were initially transcribed using Temi (San Diego, CA); French interviews were transcribed by hand. All transcriptions were cleaned and uploaded to NVivo (Burlington, MA) for qualitative analyses. Interviews were coded into themes selected according to the qualitative content analysis and number of occurrences. Because of the difference in the results of the international and local responders, the results are presented by responder type (international or national) and identified themes.

### Pilot study of training.

**Figure 1.**
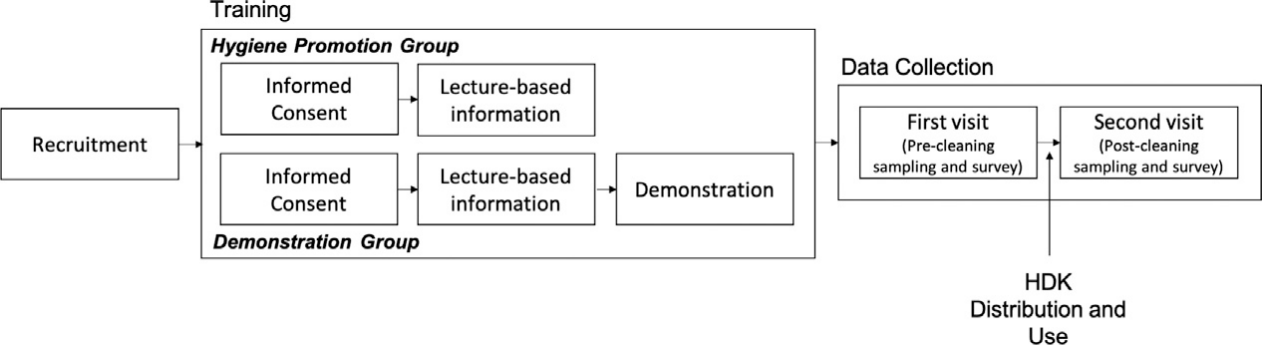
Pilot study steps.

In partnership with Clean Water for Haiti (CWH), we conducted a pilot study of HDK efficacy using different training modules in Artibonite, Haiti (Figure 1). CWH recruited 20 participants across their service area. This study focused on assessing disinfection efficacy via indicator bacteria reductions on surfaces, and we did not specifically target cholera patients during recruitment. After consenting to the study, participants were randomly assigned to receive an approximately 20-minute training session that provided lecture-based information and one fact sheet about using HDKs (Supplemental Figure S1) somewhat similar to a hygiene promotion session (hygiene promotion group) or an approximately 45-minute training session including the hygiene promotion session with demonstrations of each item provided in the HDK and time for a question-and-answer session (demonstration group). The training sessions were conducted at different times. Because recommendations vary among international agencies, we chose to base the training materials on the recommendations of the Centers for Disease Control and Prevention (CDC) and United Nations Children’s Emergency Fund (UNICEF).^10^^,^^23^ During both training sessions, it was recommended that household members prepare a 0.05% bleach solution to clean plates,^24^ dishes, and utensils, and a 0.2% bleach solution to disinfect floors and surfaces; they were also instructed to use soap to wash bedding and clothing.^23^ The number of household bleach caps per bucket of water was graphically depicted in the instruction manual (supporting information) according to the labeled concentration. We also confirmed the initial chlorine concentration of the bleach stock using the iodometric method and sodium thiosulfate.^25^ During this study, we hypothesized that participants in the demonstration group would more effectively clean their households because of the increased educational components; however, this type of training requires more logistical and financial resources.^26^^,^^27^

After the training and before HDK use, each participant household was visited by trained, local enumerators to conduct a 37-question survey about household demographics, WASH practices, and knowledge of cholera transmission pathways and to sample household surfaces. To the extent possible, selected surfaces were standardized (i.e., bedroom floor, kitchen floor, latrine floor, kitchen item, front door, curtain, water container) between households based on previous studies investigating bacterial contamination in households.^15^^,^^28^ Maps were drawn of each household to record the locations of the surfaces that were sampled and the placement of the sampling stencil. To perform sampling, a sterilized square stencil (10 × 10 cm) was placed on the surface of interest and used to define a surface area. A Sanicult™ swab (Starplex Scientific, ON, Canada) was wiped over the surface 10 times in the horizontal direction, 10 times in the vertical direction, and 10 times in the diagonal direction; then, it was returned to the 5-ml storage buffer solution. Samples were kept on ice and transported to the field laboratory for analysis within 8 hours of collection.

In the field laboratory, 250 μL of solution was spread onto thiosulfate-citrate-bile salts-sucrose (TCBS) agar (BD Difco; Becton, Dickinson and Company, Franklin Lakes, NJ) plates. After incubation at 35°C for 24 hours, medium to large yellow and blue–green colonies were recorded as suspected *Vibrio* spp., of which *V. cholerae* could have been one.^29^ Because of field testing limitations, identification and confirmation testing of colonies were not conducted to more specifically determine what *Vibrio* species formed. As such, data presented are indicative of all *Vibrio* spp., not specifically *V. cholerae*. From the remaining swab volume, 1 ml was dispensed onto a PetriFilm™ (3M, Cottage Grove, MN) and incubated at 35°C for 24 hours for simultaneous detection of *Escherichia coli* and total coliforms. The total number of colonies was calculated by multiplying the colony number by the swab volume and then dividing by the sample volume. Surface concentrations (expressed as CFU/100 cm^2^) were calculated by dividing the total number of colonies by the sampled surface area. The minimum theoretical detection limits were 20 CFU/100 cm^2^ for suspected *Vibrio* spp. and 5 CFU/100 cm^2^ for *E. coli* and total coliforms (equivalent to 1 CFU/plate). Too numerous to count (TNTC) plates were replaced by the following maximum theoretical detection limits: 5000 CFU/100 cm^2^ for suspected *Vibrio* spp. and 750 CFU/100 cm^2^ for *E. coli* and total coliforms (equivalent to 250 CFU/plate and 150 CFU/plate, respectively).^30^

After the initial survey and sample, an HDK was distributed to the household member. Each kit contained two 5-gallon buckets, one scrub brush, two cloths, one mask, one pair of plastic gloves, 1 gallon of bleach, one soap, and the instruction brochure (Supplemental Figure S1). Then, the team asked the household to use the HDK, left for a certain period of time, and returned the same day after cleaning was complete to administer a 28-question survey about participant perception of household disinfection interventions and HDK and to sample an adjacent location of the same surfaces after cleaning and disinfecting. The participants were paid for their participation (equal to approximately 1.5 days of labor in Haiti) and allowed to keep the HDK.

Data were entered in Microsoft Excel 2016 (Redmond, WA) and analyzed with R (RStudio; R Foundation for Statistical Computing, Vienna, Austria); the primary outcomes were *E. coli*, total coliforms, and *Vibrio* spp. proportion of positive surface and surface concentrations. Differences in data between groups were tested using the Fisher’s exact test or the Wilcoxon rank sum test to compare nominal and ordinal data at each sampling point. Wilcoxon’s signed-rank test was used to assess whether differences in bacterial surface concentrations were significant between the first and second visits. The degree of confidence was set to α = 0.95 for this work.

## RESULTS

### Key informant interviews.

Twenty-one people responded to the e-mails and expressed interest in participating; three were not able to schedule an interview and four did not follow-up after learning more information. Therefore, 14 KIIs were conducted between February and April 2019, including 10 performed via telephone/teleconference and four via e-mail. Among the informants, five worked at the international level (international nongovernmental organizations [INGO], United Nations agencies, or donors) and nine worked at the national level (NGO; local government, research center). Informants reported currently or previously working in Africa, Asia, and North America.

We discuss the observed differences between international-level and national-level responders regarding household disinfection knowledge. We also describe the main emergent themes of perceived barriers and facilitators of interventions, ease of implementation, perception of bleach safety, and recipient behavior change.

#### Knowledge of household disinfection.

All informants (100%; 14/14) were familiar with household spraying. Two international-level informants expressed the reasons for using household spraying were historical and based on experience and knowledge of whomever was implementing the program, with one stating: *“We try to stop household spraying, but we are there to support [local actors]. So if the stakeholders say (as it was the case last year in a lot of countries) ‘we spray because we have always done it and we will continue, no matter what type of evidence was demonstrated in recent years’, well, we are stuck.”*

Regarding HDK programs, 4 out of 5 (80%) international-level informants and 0 out of 9 (0%) national-level informants knew what an HDK was. Fewer informants (3/5 [60%] international-level and 0/9 [0%] national-level informants) had implemented HDKs. Informants who had not implemented HDKs expressed confusion regarding the difference between HDKs and hygiene kits. Therefore, the interviewer used a structured definition to explain HDK programs to all interviewees to obtain opinions about the intervention from those not familiar with it.

After learning what an HDK was, one national-level informant mentioned *“there is no need of reinventing the wheel,”* thus indicating the belief that a new intervention was not needed if household spraying was already known and implemented.

Because of the difference in intervention knowledge at the national level and international level, the results are stratified between national-level and international-level informants.

#### Perceived barriers and facilitators of household disinfection interventions.

When asked when household disinfection should occur, some informants (3/14 [21%]; all 3 international-level) highlighted that disinfection should occur within 48 hours of patient admission to prevent cholera transmission to household contacts of the case. Furthermore, 29% of informants (4/14; 3 international-level and 1 national-level) perceived that a benefit of HDKs was that household disinfection could occur more quickly than that occurring with household spraying because HDKs can be distributed to family members soon after patient admission. Additionally, because HDKs contain material that can be used multiple times, an international-level informant stated, *“We have chosen household disinfection kits due to the opportunity to have a longer-lasting effect.”*

However, 57% of informants (8/14; 4 international-level and 4 national-level) mentioned that with HDKs, it is not possible to know if household members actually used the kit. International-level informants also raised concerns about correct and consistent HDK use: “*We should be really reinforcing the systematic cleaning that needs to happen every day, multiple times a day.” “There is always a risk that disinfection was not properly done or not done at all.”* A few informants (2/14 [14%]; both international-level) highlighted that well-trained spraying teams may be less variable in their disinfection than household members using HDKs. Additionally, some informants (3/14 [21%]; 2 international-level and 1 national-level) expressed concern that households could resell or repurpose HDK items. Finally, several informants (4/14 [29%]; 1 international-level and 3 national-level) mentioned that in homes with dirt floors or high walls, household spraying is possible in places where disinfection with an HDK would be difficult.

An overall challenge noted by 28% of informants (4/14; international-level) is the lack of evidence showing that household disinfection interventions reduce caseloads during a cholera outbreak. Furthermore, an international-level informant perceived that surface transmission is only a small part of overall transmission and that the majority of infected people are asymptomatic. Another international-level informant summarized the perceptions on household disinfection efficacy, stating, *“There is always the question mark about how effective the intervention is? And is it worth it? Because if it’s not effective, what’s the point, you know?”*

#### Ease of implementation.

A total of 86% of informants (12/14; 4 international-level and 8 national-level) had experienced or felt there would be difficulties with material and human logistics during HDK distribution. Difficulties listed included standardizing kits across the same response between response organizations, item availability, quantity of materials needed, warehousing needs, program costs, training staff and beneficiaries to speak and understand appropriate local languages to distribute and use the HDK, and monitoring the intervention. One informant stated, “*With the household disinfection kits, you still need human resources that are distribution, carrying out the hygiene promotion sessions, etc.”* Additionally, a few informants (2/14 [28%]; both national-level) specifically noted that the chlorine content of bleach varies across time and by product, which can complicate HDK interventions.

Of particular note, one international-level informant with HDK implementation experience expressed that it was easier to distribute HDKs in dense urban areas where it was challenging for household spraying teams to quickly access parts of the city because of traffic. Moreover, two international-level informants who had implemented both HDK and household spraying programs acknowledged that there are high material and human logistics costs with both interventions, but that during large outbreaks, the cost of household spraying could be higher than the cost of HDK distribution. Finally, one national-level informant noted that HDK programs could be used in conflict areas where spraying teams do not have access.

#### Perception of bleach safety.

A total of 64% of informants (9/14; 3 international-level and 6 national-level informants) perceived bleach as a hazardous material, which led to concerns about the hazards of giving bleach to HDK recipients in contexts where bleach is not regularly used. One national-level informant stated, *“Bleach creates higher risk to the household or family lives because they do not understand how to use it, it is too harmful.”* In general, informants expressed concerns that bleach is harmful, difficult to dilute, easy to misuse, and could harm people if ingested. This negative perception of bleach led one international-level informant to include detergent instead of bleach in an HDK project that was implemented. Additionally, two national-level informants stated (incorrectly) that *Vibrio cholerae* bacteria would not be killed/inactivated on surfaces by chlorine in the form of bleach, and that only powder (such as the calcium hypochlorite powder commonly used to make household spraying solutions) can reduce cholera on surfaces. Furthermore, a national-level informant stated that chlorine powder is more harmful than bleach if users are not well-trained and perceived that only qualified spraying teams could perform surface disinfection.

#### Recipient behavior changes.

A total of 43% of informants (6/14; 4 international-level and 2 national-level informants) mentioned that HDK programs could lead to positive recipient behavior changes. One international-level informant stated, *“It is basically to empower the household to look after themselves. By educating them on the use of the disinfection kit, they may also have an opportunity to educate other people so they can protect themselves as well.”* Another international-level informant stated that, based on experience, positive interventions (as that informant perceived HDKs to be) were more successful than punitive actions (as that informant perceived household spraying to be). Additionally, two international-level informants felt that HDK distributions do not stigmatize people or provide a false sense of security as compared with household spraying. However, one national-level informant noted that spraying was so visible that it might increase households’ awareness of disinfection during cholera outbreaks. These perceptions were based on individual beliefs, not field data.

#### Summary and recommendations.

In summary, KIIs found low knowledge of HDKs at the national-level, a lack of evidence of the effectiveness to support decision-making, perceptions that the implementation of both HDKs and household spraying was challenging, concerns about bleach safety associated with HDK distribution, and different opinions about interventions and behavior changes. Moreover, although international-level informants who had implemented both HDKs and household spraying were more positive about HDK distribution than household spraying programs, they expressed skepticism about the effectiveness of both household disinfection interventions. This led at least one international-level informant to indicate that household disinfection was considered a lower-priority intervention than other WASH interventions in response to cholera, especially during smaller outbreaks.

Some informants (3/14 [21%]; all 3 international-level) recommended generating evidence regarding household disinfection interventions and using that evidence to develop a consensus between international-level and national-level responders. Specifically, for household spraying, informants recommended having, and using, good standard operating procedures. For HDKs, informants’ recommendations were to locally purchase products, preposition stocks in regularly affected areas, and conduct training sessions to prevent misuse of kits. Finally, informants recommended mapping transmission patterns during household disinfection, rapidly implementing household disinfection, and coordinating between partners and the affected population to develop context-specific solutions.

### Pilot study in Haiti.

During the KIIs, concerns were expressed about HDKs, including method effectiveness because of misuse or nonuse, cleaning efficacy, and difficulty conducting trainings. To address these concerns, we conducted a pilot study in February 2020 in Artibonite Department, Haiti (where bleach is regularly used) to assess HDK efficacy after two types of training.

#### Participant characteristics.

A total of 20 people participated in the training (including 11 women); 19 completed the initial pre-HDK use survey and sampling and 15 completed the second post-HDK use survey and sampling (Table 1). It was observed that households predominantly had concrete walls (17/19; 89%) and floors (18; 95%); however, brick walls (10; 53%) and dirt floors (4; 21%) were also present. Two households (10%) reported having running water, eight (42%) had electricity, 13 (68%) had a shared latrine, 4 (21%) had a private latrine, and 2 (10%) had no latrine. All participants (19/19; 100%) reported changing behavior because of previous cholera outbreaks; one participant (5%) reported a previous death in the household because of cholera and eight (42%) participants reported receiving WASH interventions as part of a previous cholera response. Two participants (10%) reported previously receiving household spraying and appreciating that service because the spraying cleaned the house, sprayers provided good advice, and other people were not scared to visit after the house was sprayed.

**Table 1:** Characteristics of participants in the hygiene promotion and demonstration groups

General information	Hygiene promotion group (*N* = 10)	Demonstration group (*N* = 9)	Total (*N* = 19)	*P* value
Participants attending the training, N	10	10	20	
First visit, N (%)	10 (100%)	9 (100%)	19 (100%)	1.00
Second visit, N (%)	6 (60%)	9 (100%)	15 (79%)	0.087
Female participants, N (%)	5 (50%)	6 (67%)	11 (58%)	1.00
Participants attended some schooling, N (%)	10 (100%)	9 (100%)	19 (100%)	1.00
Participants with male head of house able to read and write, N (%)	9 (90%)	7 (87%)	16 (89%)	1.00
Participants with female head of house able to read and write, N (%)	7 (78%)	8 (88%)	15 (83%)	0.721
Household members, mean (range)	4.2 (1–8)	5.6 (3–10)	4.8 (1–10)	0.462
Household wall material, N (%)				
Concrete	8 (80%)	9 (100%)	17 (89%)	0.290
Brick	3 (30%)	7 (78%)	10 (53%)	
Household floor material, N (%)				
Concrete	9 (90%)	9 (100%)	18 (95%)	1.00
Dirt	2 (20%)	2 (22%)	4 (21%)	
Households with running water, N (%)	1 (10%)	1 (11%)	2 (10%)	1.00
Households with latrines, N (%)				
No latrines	1 (10%)	1 (11%)	2 (10%)	0.027
Shared	6 (60%)	7 (78%)	13 (68%)	
Private	3 (30%)	1 (11%)	4 (21%)	
Household with electricity, N (%)	6 (60%)	2 (22%)	8 (42%)	0.170
Reported experience with cholera				
Participants who changed WASH habits after a cholera outbreak, N (%)	10 (100%)	9 (100%)	19 (100%)	1.00
Household with cholera death during a past outbreak, N (%)	1 (10%)	0	1 (5%)	1.00
Participants who received household spraying in the past, N (%)	1 (10%)	1 (11%)	2 (10%)	0.443
Participants who received any WASH intervention from an organization responding to the last outbreak, N (%)	2 (20%)	6 (67%)	8 (42%)	0.146
Reported cleaning practices				
**Dishwashing**				
Participants washing dishes daily, N (%)	2 (20%)	8 (89%)	10 (53%)	0.030
Participants using bleach, soap, or detergent for dishwashing, N (%)	8 (100%)	9 (100%)	17 (89%)	1.00
**Floor**				
Participants cleaning the floor daily, N (%)	7 (70%)	7 (78%)	14 (74%)	1.00
Participants using bleach or detergent to clean the floor, N (%)	8 (80%)	7 (78%)	15 (79%)	1.00
**Latrines**				
Participants cleaning the latrine daily, N (%)	1 (20%)	7 (87%)	8 (61%)	0.027
Participants using bleach or detergent to clean latrine, N (%)	4 (80%)	7 (87%)	11 (84%)	0.474
**Laundry**				
Participants washing laundry weekly, N (%)	8 (80%)	7 (78%)	15 (79%)	1.00
Participants using bleach or detergent for laundry, N (%)	10 (100%)	9 (100%)	19 (100%)	1.00

WASH = water, sanitation, and hygiene. * The *P* value (measured with Fisher’s exact test) indicates the probability that the means of the hygiene promotion group and demonstration group are equal for the assessed parameter.

Overall, differences between groups were not substantial, except for households with latrines (*P* = 0.027) and frequency in cleaning dishes and latrines (*P* = 0.030 and *P* = 0.027, respectively). There was a difference in the dropout rates before and after HDK use visits; four people (40%) in the hygiene promotion group did not use the HDK.

#### Surface contamination before and after HDK use.

The bleach concentration was tested before HDK distribution from a random sample (*N* = 6). The measured average chlorine concentration was 3.02% (standard deviation, 0.82), which was lower than the 5% advertised solution.

Ten surfaces were sampled before HDK use in all but one household, including the bedroom floor, cabinet, clothing, cooking pot, dish, door, kitchen floor, latrine floor, table, wash basin, and water container. One respondent did not have a kitchen or latrine on the premises; therefore, we only collected six samples at that location. Before cleaning, a total of 96 and 90 surfaces were sampled for the hygiene promotion group and demonstration group, respectively (Table 2). Dishes were sampled at only four households of the hygiene promotion group, and tables were not sampled in any households of the demonstration group; therefore, dishes and tables were removed from the analysis.

**Table 2:** Environmental sampling results for the hygiene promotion and demonstration groups

		Hygiene promotion group (*N* = 10)	Demonstration group (*N* = 9)	Total (*N* = 19) (*P *value*)
Surfaces sampled, N (range per household)	Before cleaning	96 (6–10 per HH)	90 (10 per HH)	186
	After cleaning	51 (5–10 per HH)	90 (10 per HH)	141
Total number of sampled surface types before/after cleaning	Bedroom floor	10/6	9/9	34
	Cabinet	9/5	9/9	32
	Clothing	10/5†	9/9	33
	Cooking pot	8/4	9/9	30
	Dish	4/3†	9/9	25
	Door	10/6	9/9	34
	Kitchen floor	9/4†	9/9	31
	Latrine floor	9/5	9/9	32
	Table	7/3	0	10
	Wash basin	10/6	9/9	34
	Water container	10/4†	9/9	32
***Escherichia coli***				
Proportion of positive surfaces, mean (range)	Before cleaning	0.26 (0–0.50)	0.33 (0.1–0.60)	0.29 (0.0–0.6) (*P* = 0.338)
	After cleaning	0.11 (0–0.28)	0.11 (0–0.30)	0.11 (0–0.30) (*P* = 1)
Mean surface concentration, CFU/100 cm^2^ (range)	Before cleaning	20.0 (< 5–445)	27.4 (< 5–> 750)	23.5 (*P* = 0.337)
	After cleaning	6.79 (< 5–40)	6.44 (< 5–50)	6.57 (*P* = 0.791)
**Total coliforms**				
Mean proportion of surfaces positive (range)	Before cleaning	0.71 (0.5–0.9)	0.81 (0.6–1.0)	0.76 (0.5–1.0) (*P* = 0.145)
	After cleaning	0.56 (0.2–0.9)	0.72 (0.3–1.0)	0.66 (0.2–1.0 (*P* = 0.214)
Mean surface concentration, CFU/100 cm^2^ (range)	Before cleaning	208 (< 5–> 750)	277 (< 5–> 750)	241 (*P* = 0.055)
	After cleaning	100 (< 5–> 750)	252 (< 5–> 750)	197 (*P* = 0.001)
***Vibrio* spp.**				
Mean proportion of surfaces positive (range)	Before cleaning	0.82 (0.5–1.0)	0.82 (0.5–1.0)	0.82 (0.5–1.0) (*P* = 0.843)
	After cleaning	0.63 (0.2–1.0)	0.67 (0.2–0.9)	0.65 (0.2–1.0) (*P* = 0.811)
Mean surface concentration, CFU/100 cm^2^ (range)	Before cleaning	1,752 (< 20–> 5,000)	1,003 (< 20–> 5,000)	1,713 (*P* = 0.705)
	After cleaning	1,672 (< 20–> 5,000)	635 (< 20–> 5,000)	768 (*P* = 0.356)

CFU = colony-forming unit; HH = household.

* The *P* value (measured with the Wilcoxon rank-sum test) indicates the probability that the means of the hygiene promotion group and the demonstration group are equal for the assessed parameter.

† Surface was missing/not accessible during follow-up visit sampling.

**Figure 2.**
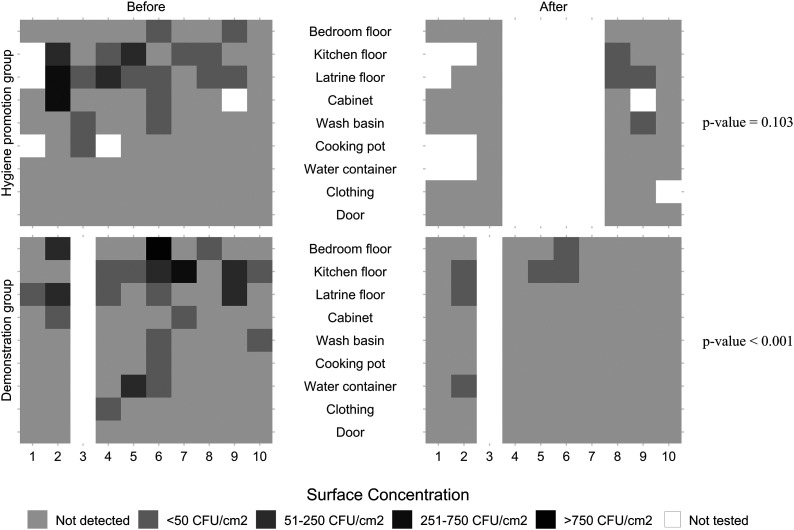
*Escherichia coli* surface concentrations before and after household disinfection kit (HDK) use. The *P* value (measured using the Wilcoxon’s signed-rank test) indicates the probability that the precleaning and postcleaning surface concentration means are equal. Adapted from Gallandat et al. (2020) for measured bacteria concentrations on surfaces involved in household spraying programs.

Before HDK use, the average proportions of surfaces positive for *E. coli* per household were 0.26 and 0.33 for the hygiene promotion group and demonstration groups, respectively, with mean surface concentrations of 20.0 CFU/cm^2^ for the hygiene promotion group and 27.4 for the demonstration group (Table 2). For total coliforms, the average proportions of surfaces positive per household were 0.71 and 0.81 for the hygiene promotion and demonstration groups, respectively, with mean surface concentrations of 208 and 277 CFU/cm^2^. *Vibrio* spp. were found on an average of 82% of the surfaces for both groups, with mean surface concentrations of 1,752 and 1,003 CFU/cm^2^ for the hygiene promotion and demonstration groups, respectively. Before the use of the HDK, there was no significant difference between the training groups in the proportion of contaminated surfaces or the initial mean surface contaminations of *E. coli*, total coliforms, or *Vibrio* spp. In both groups, the most contaminated surfaces were the floors in the bedroom, latrine, and kitchen (Figure 2 and Supplemental Figure S2).

After HDK use, a total of 51 and 90 surfaces were sampled in households of the hygiene promotion and demonstration groups, respectively (Table 2). The sampling size was lower because four participants (40%) in the hygiene promotion group did not use the HDK before the return visit. Additionally, some surfaces were not accessible during the post-HDK use visit because areas were locked. After cleaning, an average of 11% of surfaces/households were positive for *E. coli*, with mean surface concentrations of 6.79 CFU/cm^2^ for the hygiene promotion group and 6.44 for the demonstration group (Table 2). For total coliforms, averages of 56% and 72% of surfaces/households were positive, with mean surface concentrations of 100 CFU/cm^2^ and 252 CFU/cm^2^ for the hygiene promotion and demonstration groups, respectively. For *Vibrio* spp., averages of 63% and 67% surfaces/households in the hygiene promotion and demonstration groups were positive, respectively, with mean surface concentrations of 1,672 CFU/cm^2^ and 635 CFU/cm^2^. After HDK use, there was no significant difference between the training groups in the proportion of contaminated surfaces or the mean surface contamination of *E. coli* or *Vibrio* spp. However, the mean total coliform surface concentration was higher (*P* = 0.001) after HDK use in the households of the demonstration group than in the households of hygiene promotion group. The kitchen floors remained the most contaminated surfaces (Figure 2 and Supplemental Figure S2).

There was a significant reduction in *Vibrio* spp. on surfaces after the use of the HDK for both groups (*P* = 0.012 hygiene promotion group; *P* < 0.001 demonstration group). There was also a significant reduction in *E. coli* concentrations after HDK use for the demonstration group only (*P* = 0.103 hygiene promotion group; *P* < 0.001 demonstration group) (Figure 2 and Supplemental Figure S2). Additionally, there was no significant reduction in total coliforms after HDK use for either group (*P* = 0.056 hygiene promotion group; *P* = 0.266 demonstration group). There was no influence on the results related to the time that had passed between training and HDK use, surveying, and sampling.

#### Self-reported use of HDK and HDK perception.

A total of 73% of participants perceived the training as very useful (40% hygiene promotion group; 78% demonstration group), with the remainder stating the training was useful (Table 3). Participants liked the HDK because of the training (67% hygiene promotion group; 100% demonstration group), it contained useful items (67% both groups), it was easy to use (17% hygiene promotion group; 22% demonstration group), and it was free (17% hygiene promotion group; 11% demonstration group). Only one participant from the hygiene promotion group (17%) did not like the HDK and stated that larger quantities were needed. Four participants from the demonstration group (44%) disliked the HDK because they could not buy refill materials (33%) or it was difficult to use (11%) (Table 3).

**Table 3:** Self-reported use of HDK materials for the hygiene promotion and demonstration groups

	Hygiene promotion group (*N* = 6)	Demonstration group (*N* = 9)	Total (*N* = 15)
**Soap**			
Participants reported using the soap, N (%)	1 (17%)	3 (33%)	4 (27%)
**Bleach**			
Participants reported using the bleach, N (%)	6 (100%)	9 (100%)	15 (100%)
Participants reported using a 0.05% solution for the dishes, N (%)	2 (33%)	8 (89%)	10 (67%)
Participants reported using a 0.2% solution to clean, N (%)			
Floors	3 (50%)	8 (89%)	11 (73%)
Surfaces and doors	1 (17%)	8 (89%)	9 (60%)
Latrine	2 (33%)	7 (78%)	9 (60%)
**Perception of the disinfection kit**			
Participants who thought the training was very useful, N (%)	4 (67%)	7 (78%)	11 (73%)
Participants who thought the training was useful, N (%)	2 (33%)	2 (22%)	4 (27%)
Participants who liked the HDK, N (%)			
Because of good training	4 (67%)	9 (100%)	13 (87%)
Because the kit contained many useful items	4 (67%)	6 (67%)	10 (67%)
Because it was easy to use	1 (17%)	2 (22%)	3 (20%)
Because it was free	1 (17%)	1 (17%)	2 (13%)
Participants who disliked the HDK, N (%)			
Because I cannot buy refills	0	3 (33%)	3 (20%)
Because it is difficult to use	0	1 (11%)	1 (7%)
Because I need larger quantities	1 (17%)	0	1 (7%)
Bad training	0	0	0
Participants who would prefer to receive an HDK (vs. HS), N (%)	4 (67%)	9 (100%)	13 (87%)
Because it saves time and I can often clean my house using the kit	1 (17%)	4 (44%)	5 (33%)
Because people can be neglectful when doing a job for you	1 (17%)	2 (22%)	3 (20%)
Because it is my own responsibility to clean the house	1 (17%)	2 (22%)	3 (20%)
Because I can use the kit on all areas of my house	1 (17%)	1 (11%)	2 (13%)
Because participants would prefer to receive HS (vs. HDK), N (%)	2 (33%)	0 (0%)	2 (13%)

HDK = household disinfection kit; HS = household spraying.

Finally, all participants from the demonstration group and 67% of participants from the hygiene promotion group reported that if they could choose, they would prefer using an HDK rather than household spraying (Table 3). Reasons for this preference included that households do not have to wait for the spraying team and could use the HDK more than once (33%), strangers could be neglectful when disinfecting (20%), it is a household’s own responsibility to clean the house (20%), or a household could use the kit items on all areas of the house (13%). Two participants (33%) from the hygiene promotion group mentioned they would prefer household spraying because sprayers would be better trained to remove bacteria or because it would be difficult to find HDK items. These two participants did not self-report having previously experienced household spraying.

## DISCUSSION

To understand why HDKs were not being implemented, and to identify facilitators of, barriers to, and recommendations for implementing household disinfection interventions to prevent intrahousehold cholera transmission, we conducted 14 KIIs with international-level and national-level implementers and one pilot study in Haiti that assessed HDK use after training in 20 households. We observed the following: differences in knowledge, perceptions, and experiences with household disinfection between international-level and national-level informants; evidence gaps for household spraying and HDKs led responders to use other criteria to select household disinfection interventions; high concentrations of *E. coli*, total coliforms, and *Vibrio* spp. on bedroom, latrine, and kitchen floors before disinfection; and variable bacterial susceptibility to HDK use, with significant reductions in *Vibrio *spp. across both training groups and increased *E. coli* reductions in the demonstration group.

Despite international recommendations regarding household disinfection interventions,^16^^,^^20^ we observed there is little consensus, especially between responders at the international and national levels. This is partially because of the lack of evidence and knowledge of household disinfection interventions. Among the 14 KII informants, only the four international-level informants knew of both household spraying and HDK distribution interventions, and only three had implemented both interventions. Conversely, no national-level informants knew what an HDK was and did not consider HDKs a valid intervention option after explanation. This represents a disparity between what intervention is recommended at the international level and what intervention is implemented at the national level.

The main benefits of HDKs, as perceived by international-level informants, were the positive impact on the behavior change of beneficiaries, not depending on spraying teams to arrive at a household, and the possibility for households to repeat disinfection. During their guidance regarding managing cholera outbreaks, two international response organizations have also cited the possibility for HDKs to have a positive impact on beneficiaries’ awareness of disinfection,^17^^,^^31^ and another organization has indicated the benefits of a household using the kit multiple times.^18^ During the pilot study, household respondents also mentioned that using an HDK allowed them to repeat disinfection. They added that it is the household’s responsibility to clean the house and that nonhousehold members can be neglectful. The main benefits of household spraying, as perceived by both international-level and national-level informants, were that it enables systematic disinfection on any type of surface and mapping of the outbreak. Although informants summarized that each intervention has specific advantages, they highlighted that there are more drawbacks than benefits for both interventions. In particular, informants expressed concerns with household exposure to bleach provided in HDKs and the gap in evidence supporting either intervention. Additionally, both interventions are considered difficult to implement, with high logistical and human resource costs. Overall, responders justified the implementation of both HDKs and household spraying because they believe these interventions are beneficial (e.g., possible beneficiary hygiene behavior change or context-specific familiarity with intervention) despite the evidence gaps and drawbacks of implementing household disinfection during cholera outbreaks.

Before disinfection during the pilot study, we observed the most contaminated surfaces with *E. coli*, total coliforms, and *Vibrio* spp. were the bedroom floors, latrine floors, and kitchen floors. This result is consistent with previous evaluations of indicator bacteria on household surfaces.^15^^,^^28^ After HDK use, *E. coli* and *Vibrio* spp. were significantly reduced on household surfaces, with average reductions from 23.5 CFU/cm^2^ to 6.57 for *E. coli* and from 1,713 CFU/cm^2^ to 768 for *Vibrio* spp. However, total coliforms were not significantly reduced. Therefore, wiping using HDK materials can effectively remove some bacteria (*Vibrio* spp. and *E. coli*) from household surfaces, but bacteria present on household surfaces exhibit variable tolerance for wiping and/or chlorine at the concentrations tested.^32^ We recommended using 0.2% and 0.05% chlorine solutions, which are different than the concentrations used during previous work^15^^,^^22^ but are recommended by the CDC^24^ and UNICEF^31^ and are easy to prepare using a household chlorine solution.^33^ After HDK use, in almost all households, there were still some contaminated surfaces. This is consistent with residual contamination observed after household spraying by programs without systematic spraying procedures.^15^

The pilot study also showed a positive HDK perception by participants, with 87% of participants reporting liking HDKs and 60% to 73% of participants who reported using the 0.05% and 0.2% concentration solutions to clean being trained. We observed a higher attrition rate during the first training session (4/10 in the hygiene promotion group); however, before the second training session, the research team made slight adjustments to emphasize that participants were expected to use the HDK as part of study enrollment. It is not clear if other nonmonetary incentives could increase the use of HDKs because the motivation for learning and performing disinfection might be different in other contexts^34^ (e.g., having a household member with cholera).

The results also showed there were two main differences between training groups. First, self-reported HDK use was higher for participants who attended the demonstration session; 89% (compared with 33% of participants in the hygiene promotion group) reported using the appropriate solution to disinfect dishes, and 78% to 89% (compared with 17–50%) mentioned using a 0.2% solution to clean the floor, latrine, and other surfaces. Additionally, surfaces disinfected by participants who attended the demonstration session had significant reductions in both *E. coli* and *Vibrio* spp., whereas only *Vibrio* spp. were significantly reduced on surfaces in households of the hygiene promotion group. These results highlight the need for appropriate and sufficient training for HDK recipients.

There were some limitations to this work. There was a small sample size of key informants with knowledge of both household disinfection interventions. There was a high loss to follow-up in the hygiene promotion group in the pilot study but not in the demonstration group that may have been attributable to a reminder from the trainer that participation entailed utilizing the kit. The pilot study was not conducted during a cholera outbreak. We could not confirm the presence of *V. cholerae* among *Vibrio* spp. because of field testing limitations, although during a noncholera outbreak this was not a crucial outcome. There were possible biases in responses from KII informants related to personal intervention experiences and responses/behaviors of participants in the household pilot study as a result of being paid to participate. We were unable to control the concentration of the locally available chlorine solution. We were unable to sample household-prepared cleaning solutions to confirm what concentrations of chlorine solution were used. The household sampling was performed only once after HDK use, and we did not assess repeated HDK use over time or loss of training information. However, we do not feel that these limitations impacted our research conclusions.

Although studies have shown that chlorine reduces bacteria (including *V. cholerae*) on both nonporous and porous household surfaces,^35^ challenges related to the maintenance of chlorine concentrations, volumes of solution used, disinfection protocols, and chlorine application methods remain.^15^^,^^22^ A previous field evaluation of household disinfection by spraying and laboratory efficacy study of chlorine disinfection against *V. cholerae* indicated the importance of ensuring that surfaces are thoroughly wetted with disinfectant to achieve microbial reduction.^15^^,^^22^ Therefore, future research specifically focusing on HDK distribution should quantify the in vivo effectiveness of HDK use to reduce surface contamination during a cholera outbreak, assess the context-specific likelihood of HDK use by beneficiaries, assess the effect of HDK use over time on household contamination levels and behavior change, and address how to adapt training across different contexts. Additionally, household spraying and HDK interventions should be compared in terms of cost-effectiveness when implementing alone and in conjunction with other WASH programs to identify opportunities to make household disinfection logistically more efficient.

Overall, the results of this study further the understanding of responder perception of HDK interventions and pilot data showing that HDKs can be used effectively by households after training to reduce microbial surface contamination. Therefore, additional studies and assessments of HDK programs are necessary. Our work highlights the need for evidence to align household disinfection recommendations as well as dissemination of knowledge and training to responders and affected populations to prevent inter-household cholera transmission. Despite international recommendations that household spraying programs should be stopped and HDK programs should be implemented, the reality is that household spraying continues to be the favored household disinfection intervention among national-level staff. Furthermore, the lack of knowledge of HDKs among national-level staff in this study and the absence of any HDK program for us to evaluate during the 3 years of our research timeframe indicate a significant gap between current policy and cholera response programming. Most importantly, there is a fundamental need for research to inform the prioritization of WASH interventions in response to cholera and to provide a better understanding of how much surface-mediated cholera transmission contributes to within-household transmission.

## Supplemental figures


Supplemental materials

Supplemental materials
