# Supplementary material for: Household Disinfection Interventions to Prevent Cholera Transmission: Facilitators, Barriers, Training, and Evidence Needs
Source: Am J Trop Med Hyg. 2021 Jul 6;105(3):611–21. doi: 10.4269/ajtmh.20-1314 (PMC8592341; doi:10.4269/ajtmh.20-1314)
Supplement: Supplementary file 1 [file tpmd201314.SD1.pdf]

<table>
<tr><th>1. Make the bleach and prepare the soap</th><th colspan="3">2. Get the items</th></tr>
<tr><td rowspan="3">Bleach solutions must be prepared daily.

Prepare the bleach solutions in a well-ventilated area.

Avoid direct contact with skin and eyes.</td><td colspan="2">Material to clean
The kit will include the following items that will be bought locally:
- 2 buckets (5 gallons or 20L)
- 1 scrub-brush
- 2 cloths
- 1 mask and plastic gloves to protect the safety of the cleaner
- 6 L of bleach for cleaning
- 1 soap or disinfectant for washing clothes and bedding</td><td>Safety

For safety reasons, use gloves and a mask when washing with bleach.</td></tr>
<tr><th colspan="3">3. Clean the house</th></tr>
<tr><th>Kitchen and eating area</th><th>Bedroom</th><th>Latrine</th></tr>
<tr><td>Prepare a concentrated bleach solution: ➔
- Take a clean bucket
- Fill the bucket with water
- Take … caps of household bleach
- Pour in the bucket full of water</td><td>Household surfaces of the kitchen and the eating area are cleaned regularly
- Wear gloves and mask
- With the brush and the cloths, clean the kitchen and the eating area with the solution</td><td>Bedroom surfaces are cleaned regularly

- Wear gloves and mask
- With the brush and the cloths, clean the bedroom with the solution</td><td>Latrine surfaces are cleaned regularly

- Wear gloves and mask
- With the brush and the cloths, clean the latrine with the solution</td></tr>
<tr><td>Prepare a diluted bleach solution: ➔
- Take a clean bucket
- Fill the bucket with water
- Take … caps of household bleach
- Pour in the bucket full of water</td><td>Plates, dishes and utensils are cleaned regularly
- Clean plates, dishes and utensils with the solution, the brush and the cloth.
- Let it air dry</td><td>-</td><td>-</td></tr>
<tr><td>With soap and water ➔</td><td>-</td><td>- Wash bedding, clothing, and diapers with soap at least 30 meters away from all bodies of water.
- Air dried before reuse.
- Dispose wastewater in latrine or sanitation system
- Wash your hands afterwards</td><td>-</td></tr>
</table>

1. Make the bleach and prepare the soap

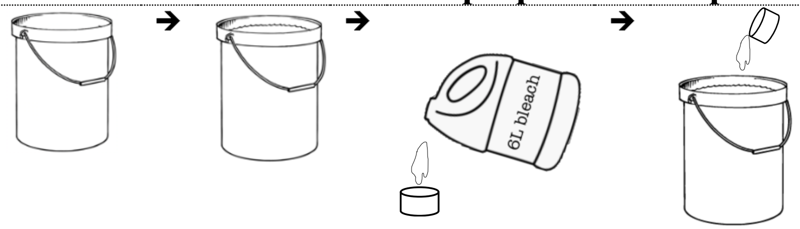

2. Get the items

Material to clean

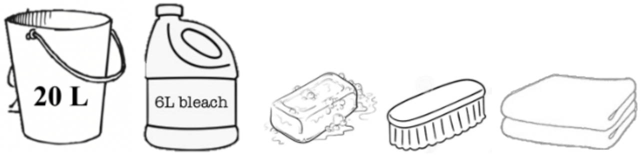

Safety

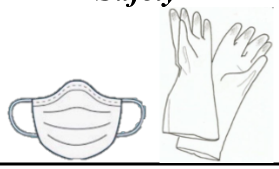

3. Clean the house

		Kitchen and eating area	*Bedroom*	*Latrine*
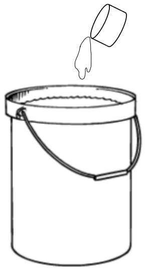	x 20 ➔	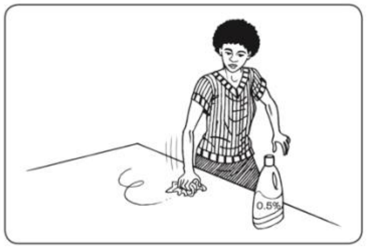	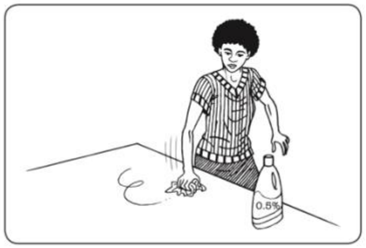	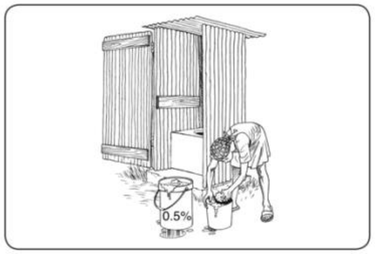
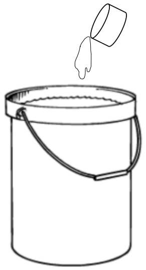	x 5 ➔	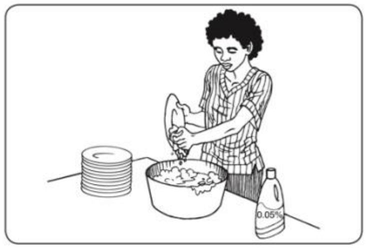	-	-
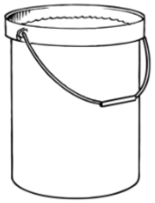	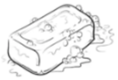 ➔	-	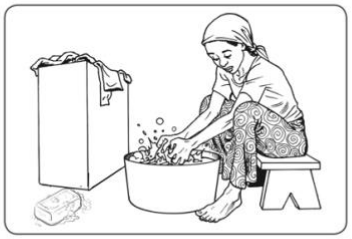	-

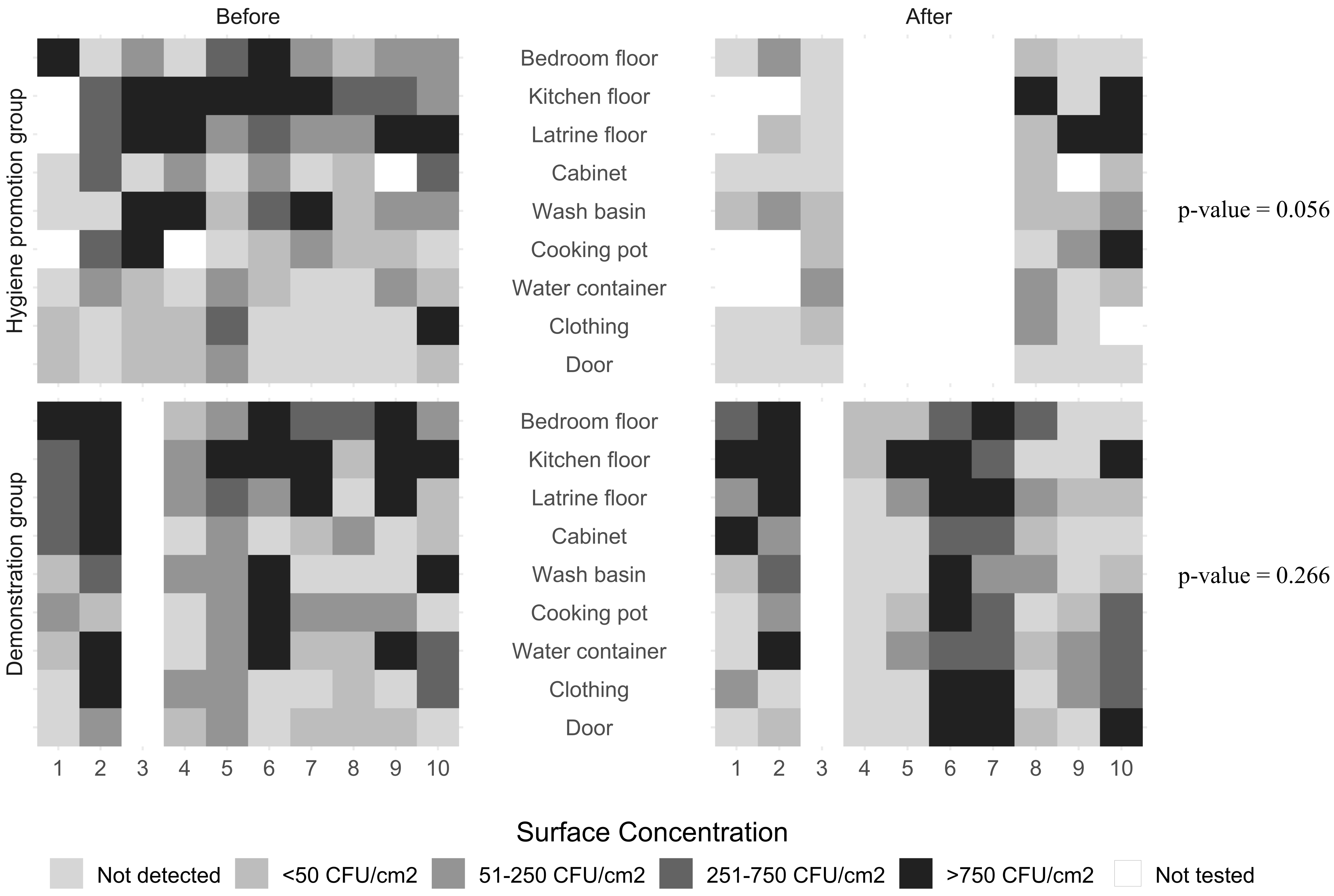

Before
After
Hygiene promotion group
Demonstration group
Bedroom floor
Kitchen floor
Latrine floor
Cabinet
Wash basin
Cooking pot
Water container
Clothing
Door
p-value = 0.056
p-value = 0.266
1 2 3 4 5 6 7 8 9 10
Surface Concentration
Not detected
<50 CFU/cm2
51-250 CFU/cm2
251-750 CFU/cm2
>750 CFU/cm2
Not tested

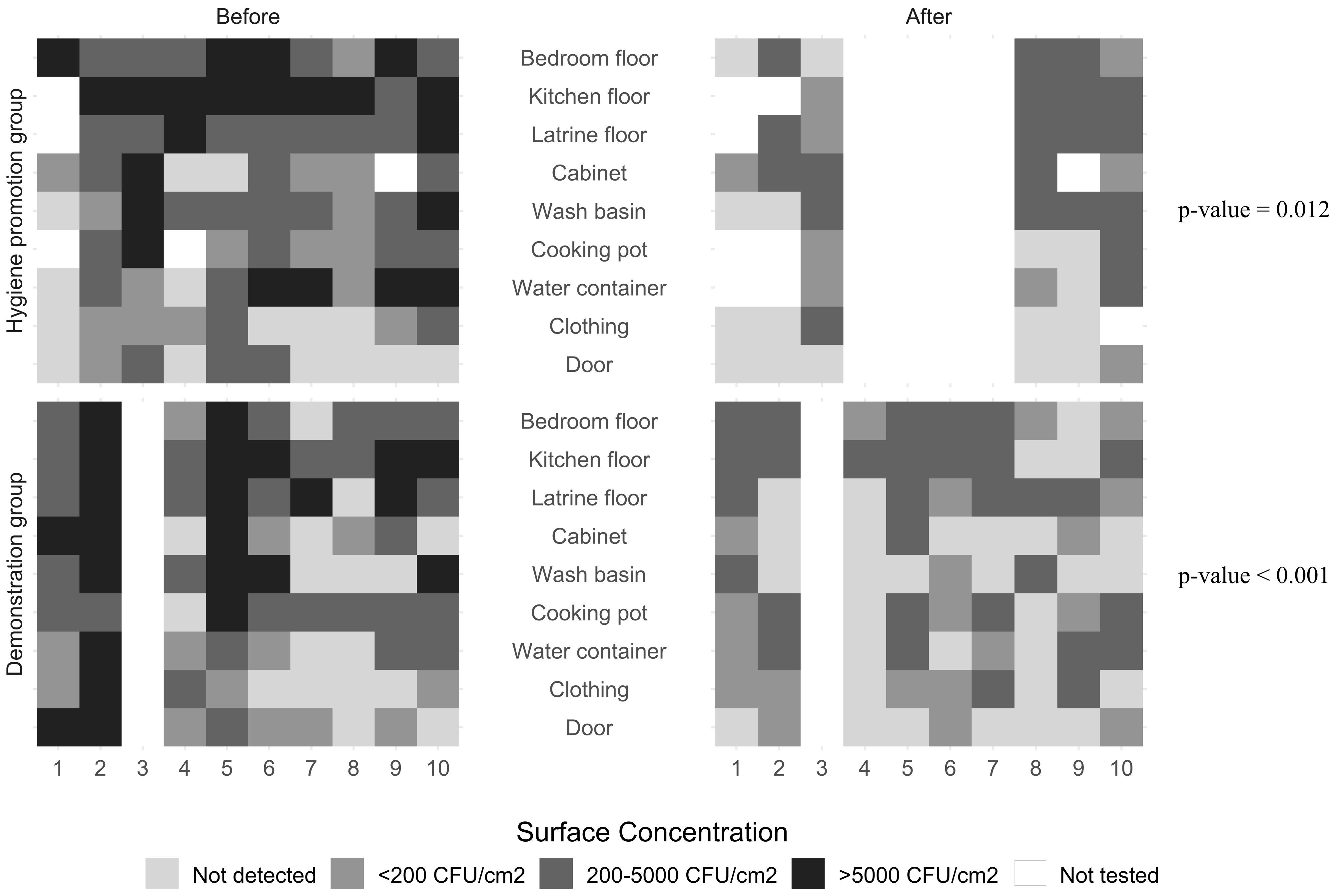
Before
After
Hygiene promotion group
Demonstration group
Bedroom floor
Kitchen floor
Latrine floor
Cabinet
Wash basin
Cooking pot
Water container
Clothing
Door
p-value = 0.012
p-value < 0.001
1 2 3 4 5 6 7 8 9 10
Surface Concentration
Not detected
<200 CFU/cm2
200-5000 CFU/cm2
>5000 CFU/cm2
Not tested
