# Supplementary material for: Household Disinfection Interventions to Prevent Cholera Transmission: Facilitators, Barriers, Training, and Evidence Needs
Source: Am J Trop Med Hyg. 2021 Jul 6;105(3):611–21. doi: 10.4269/ajtmh.20-1314 (PMC8592341; doi:10.4269/ajtmh.20-1314)
Supplement: Supplementary file 2 [file tpmd201314.SD2.docx]

**Figure S1: Fact sheet on using HDKs.** Each HDK contained an instruction manual in Haitian Kreyòl and in French.

| **1. Make the bleach and prepare the soap** | | | **2. Get the items** | | |
| --- | --- | --- | --- | --- | --- |
| Bleach solutions must be prepared daily.  Prepare the bleach solutions in a well-ventilated area.  Avoid direct contact with skin and eyes. | | | ***Material to clean***  The kit will include the following items that will be bought locally:   - 2 buckets (5 gallons or 20L) - 1 scrub-brush - 2 cloths - 1 mask and plastic gloves to protect the safety of the cleaner - 6 L of bleach for cleaning - 1 soap or disinfectant for washing clothes and bedding | | ***Safety***  For safety reasons, use gloves and a mask when washing with bleach. |
|  |  |  | **3. Clean the house** | | |
|  |  |  | ***Kitchen and eating area*** | ***Bedroom*** | ***Latrine*** |
| Prepare a ***concentrated*** bleach solution:   - Take a clean bucket - Fill the bucket with water - Take … caps of household bleach - Pour in the bucket full of water | 🡺 | | Household surfaces of the kitchen and the eating area are cleaned regularly   - Wear gloves and mask - With the brush and the cloths, clean the kitchen and the eating area with the solution | Bedroom surfaces are cleaned regularly   - Wear gloves and mask - With the brush and the cloths, clean the bedroom with the solution | Latrine surfaces are cleaned regularly   - Wear gloves and mask - With the brush and the cloths, clean the latrine with the solution |
| Prepare a ***diluted*** bleach solution:   - Take a clean bucket - Fill the bucket with water - Take … caps of household bleach - Pour in the bucket full of water | | 🡺 | Plates, dishes and utensils are cleaned regularly   - Clean plates, dishes and utensils with the solution, the brush and the cloth. - Let it air dry | - | - |
| With soap and water | | 🡺 | - | - Wash bedding, clothing, and diapers with soap at least 30 meters away from all bodies of water. - Air dried before reuse. - Dispose wastewater in latrine or sanitation system - Wash your hands afterwards | - |

| **1. Make the bleach and prepare the soap** | | | | | | | | | **2. Get the items** | | | | |  |
| --- | --- | --- | --- | --- | --- | --- | --- | --- | --- | --- | --- | --- | --- | --- |
| 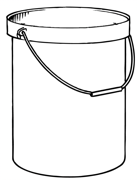 | 🡺 | 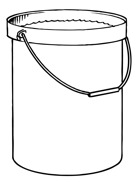 | 🡺 |  | | 🡺 | | ****** | ***Material to clean***  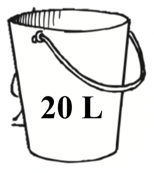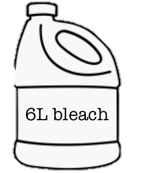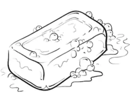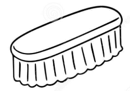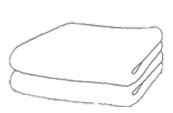 | | | | ***Safety***  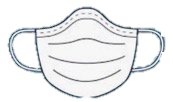 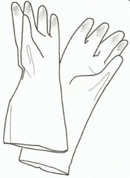 | |
|  |  |  |  |  |  |  |  |  | **3. Clean the house** | | | | | |
|  |  |  |  |  |  |  |  | ***Kitchen and eating area*** | | | ***Bedroom*** | | ***Latrine*** | |
|  | | | | | x 20 | 🡺 | | 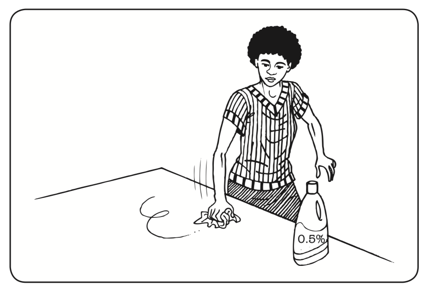 | | | 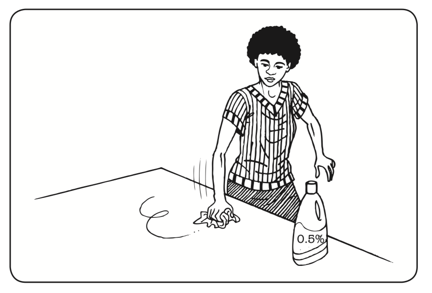 | | 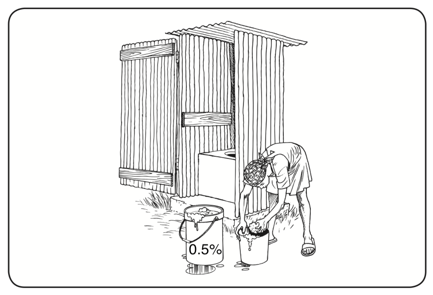 | |
|  | | | | | x 5 | 🡺 | | 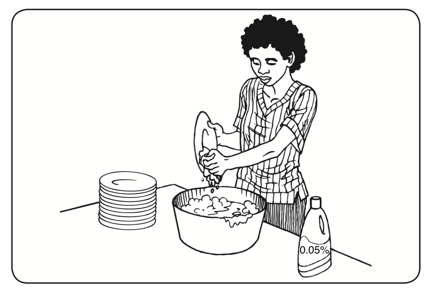 | | | - | | - | |
| 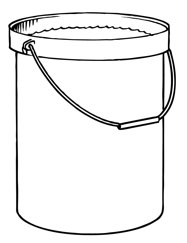 | | | | | 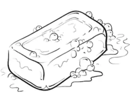 | | 🡺 | - | | 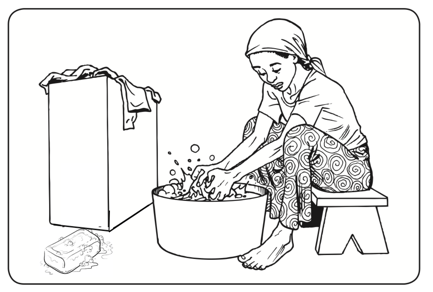 | | - | |  |

**Figure S2.** Total coliforms (top) and *Vibrio* spp*.* (bottom) surface concentrations, before and after HDK use. Figure style adapted from Gallandat et al. (2020) for measured bacteria concentration on surfaces in household spraying programs.

| **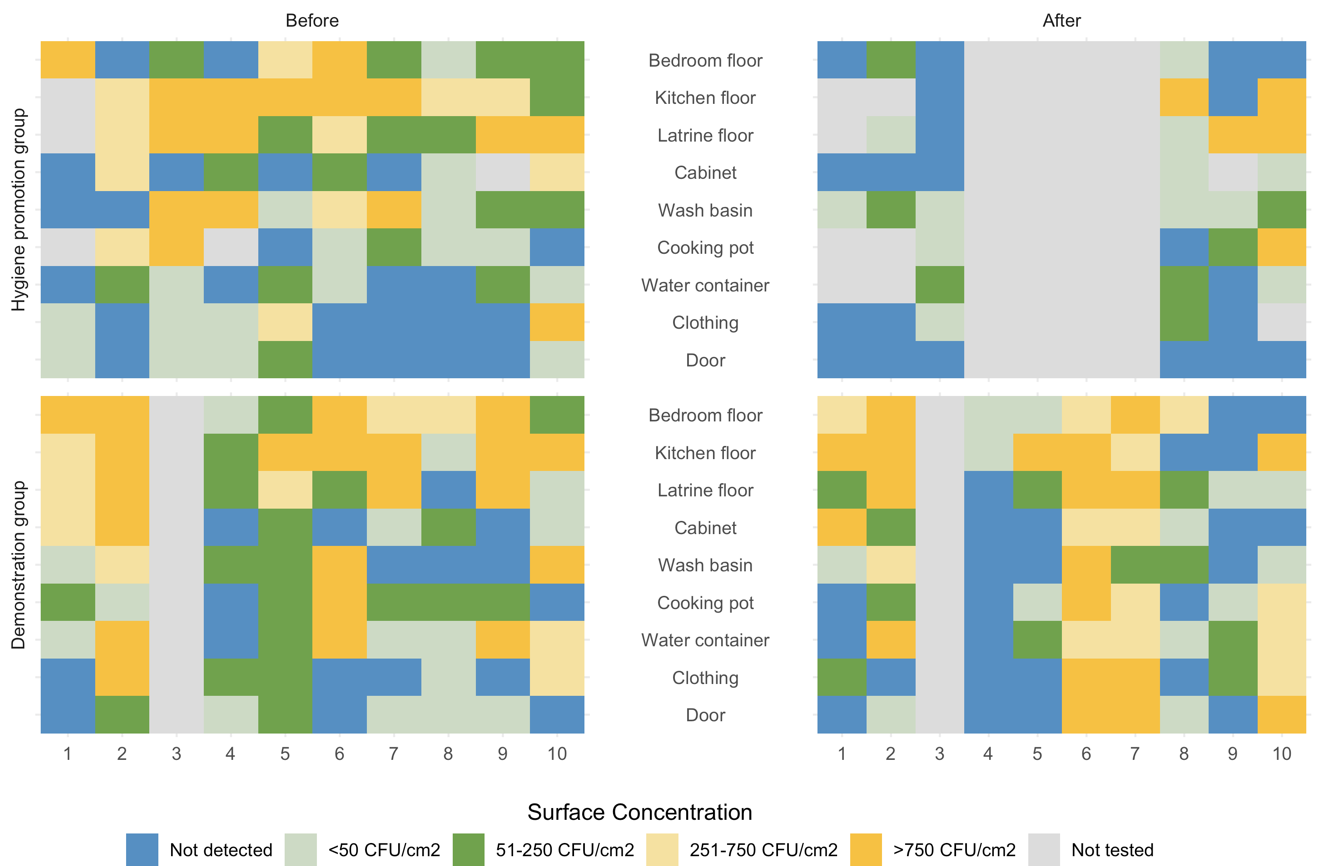** | p-value=0.056  p-value=0.266 |
| --- | --- |

| **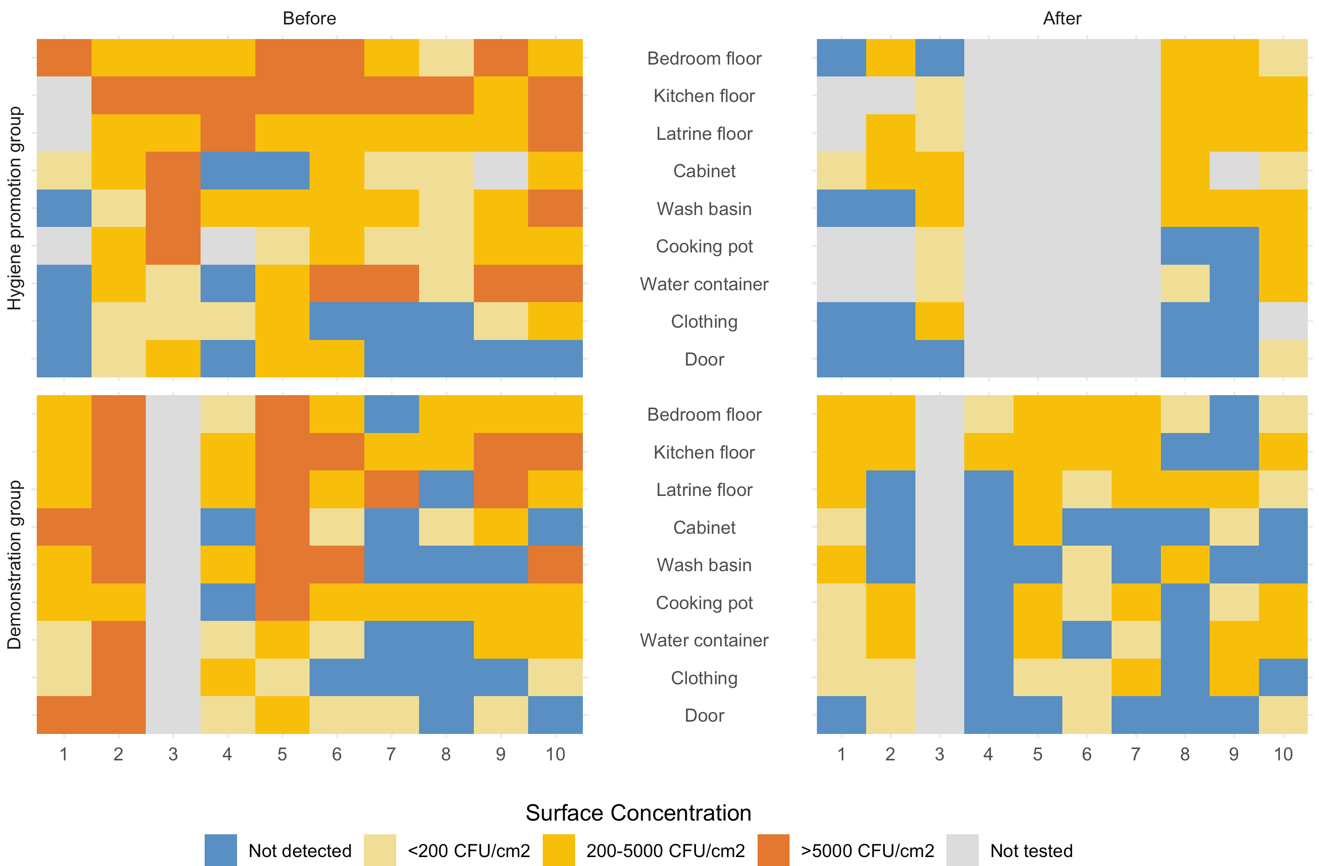** | p-value=0.012  p-value<0.001 |
| --- | --- |
